# Water Stress Modulates Soybean Aphid Performance, Feeding Behavior, and Virus Transmission in Soybean

**DOI:** 10.3389/fpls.2016.00552

**Published:** 2016-04-27

**Authors:** Punya Nachappa, Christopher T. Culkin, Peter M. Saya, Jinlong Han, Vamsi J. Nalam

**Affiliations:** Department of Biology, Indiana University-Purdue University Fort WayneFort Wayne, IN, USA

**Keywords:** drought, flooding, soybean aphid, soybean mosaic virus, amino acids, abscisic acid, salicylic acid, jasmonic acid

## Abstract

Little is known about how water stress including drought and flooding modifies the ability of plants to resist simultaneous attack by insect feeding and transmission of insect-vectored pathogen. We analyzed insect population growth, feeding behaviors, virus transmission, and plant amino acid profiles and defense gene expression to characterize mechanisms underlying the interaction between water stress, soybean aphid and aphid-transmitted, *Soybean mosaic virus*, on soybean plants. Population growth of non-viruliferous aphids was reduced under drought stress and saturation, likely because the aphids spent less time feeding from the sieve element on these plants compared to well-watered plants. Water stress did not impact population growth of viruliferous aphids. However, virus incidence and transmission rate was lowest under drought stress and highest under saturated conditions since viruliferous aphids took the greatest amount time to puncture cells and transmit the virus under saturated conditions and lowest time under drought stress. Petiole exudates from drought-stressed plants had the highest level of total free amino acids including asparagine and valine that are critical for aphid performance. Aphids did not benefit from improved phloem sap quality as indicated by their lower densities on drought-stressed plants. Saturation, on the other hand, resulted in low amino acid content compared to all of the other treatments. Drought and saturation had significant and opposing effects on expression of marker genes involved in abscisic acid (ABA) signaling. Drought alone significantly increased expression of ABA marker genes, which likely led to suppression of salicylic acid (SA)- and jasmonic acid (JA)-related genes. In contrast, ABA marker genes were down-regulated under saturation, while expression of SA- and JA-related genes was up-regulated. We propose that the apparent antagonism between ABA and SA/JA signaling pathways contributed to an increase in aphid densities under drought and their decrease under saturation. Taken together, our findings suggests that plant responses to water stress is complex involving changes in phloem amino acid composition and signaling pathways, which can impact aphid populations and virus transmission.

## Introduction

Water stress including drought and flooding is the most important factor affecting the outcome of plant- herbivore and plant–pathogen interactions (Rosenzweig et al., 2001). There is a wealth of information related to performance of different aphid species on drought-stressed plants (Huberty and Denno, 2004). For example, studies have reported positive (Khan et al., 2010; Mewis et al., 2012), negative (McVean and Dixon, 2001; Hale et al., 2003), and neutral (Salas and Corcuera, 1991; Pons and Tatchell, 1995) effects of drought on aphid performance. Drought stress can have equally complex consequences on plant–pathogen interactions. Most studies report reduced disease resistance in plants under drought stress, but there is considerable variation in the outcomes (Mauch-Mani and Mauch, 2005; Fujita et al., 2006; Asselbergh et al., 2008). For instance, drought stress increased the development of Pierce’s disease symptoms caused by the bacterial pathogen, *Xylella fastidiosa* in grapevine (Thorne et al., 2006). Conversely, drought-stressed plants were shown to be resistant to certain pathogens. In tomato, for example drought reduced gray mold infection caused by *Botrytis cinerea* by 50% and also suppressed spread of the powdery mildew fungus, *Oidium neolycopersici* (Achuo et al., 2006). In contrast, there is limited information on the influence of flooding or saturation on plant–herbivore and plant–pathogen interactions. Studies have found that populations of the generalist aphid, *Myzus persicae* was reduced under saturated conditions, whereas the specialist aphid *Brevicoryne brassicae* was less affected by saturation (Khan et al., 2010; Mewis et al., 2012). Flooding has shown to benefit epidemics and prevalence of several fungal pathogens in corn, soybean, alfalfa, and wheat (Rosenzweig et al., 2001).

To date most studies have focused on understanding the direct effects of drought stress on plants and herbivores, but it is also important to understand indirect effects of drought on species interactions. There is accumulating information on the impact of drought stress on insect-transmitted pathogens. Krugner and Backus (2014) showed that drought stress reduced the frequency of probes by the glassy winged sharpshooter, *Homalodisca vitripennis* that are critical for transmission and spread of *Xylella fastidiosa*, bacterial pathogen of Pierce’s diseases in grapes. In contrast, drought stress enhanced the plant-to-plant movement of bird cherry-oat aphids, *Rhopalosiphum padi* thereby increasing the proportion of plants infected with *Barley yellow dwarf virus* (BYDV) (Smyrnioudis et al., 2000). More recently, Davis et al. (2015b) showed that *R. padi* feeding on drought-stressed BYDV-infected plants had greater population growth rate compared to non-infected water stressed plants suggesting that virus infection helps aphids perform better on suboptimal plants. In addition, the authors found that BYDV infection in wheat increased total phytohormone concentration specifically that of SA in a time-dependent manner, which may play a role in plant resistance to drought (Davis et al., 2015a).

Plant responses during drought stress are mainly regulated by the stress hormone, ABA, which results in the activation of transcription factors and downstream functional genes that re-establish homeostasis in the plant (Finkelstein et al., 2002; Ramanjulu and Bartels, 2002; Asselbergh et al., 2008; Urano et al., 2009; Harb et al., 2010; Bostock et al., 2014). In addition to its function in abiotic stress, ABA also impacts plant resistance to pathogens and herbivores (Asselbergh et al., 2008; Studham and MacIntosh, 2013; Guo et al., 2016). There is evidence for both antagonistic and synergistic interactions between ABA and hormonal pathways that regulate plant defenses. ABA can suppress SA-mediated defenses (Mohr and Cahill, 2003, 2007; Thaler and Bostock, 2004; Asselbergh et al., 2008) and plant susceptibility to pathogens can increase following exogenous applications of ABA (McDonald and Cahill, 1999; Asselbergh et al., 2007, 2008). In certain instances, exogenous application of ABA can have the opposite effect on SA-mediated defenses resulting in increased resistance to pathogens (Ton and Mauch-Mani, 2004; Wiese et al., 2004; Melotto et al., 2006). The antagonism of SA-mediated defenses by ABA may be explained in part by the positive effect of ABA on JA biosynthesis (Adie et al., 2007). In addition, changes in proteins in ethylene (ET) and JA signaling were observed in maize leaves during drought (Bonhomme et al., 2012). There is also evidence that aphid feeding increases ABA levels in several crop species including, barley (Casaretto et al., 2004), eggplant, squash (El-Khawas and El-Khawas, 2008), and soybeans (Studham and MacIntosh, 2013). In soybeans, it has been hypothesized that soybean aphids induce ABA expression as a decoy strategy to suppress SA- and JA-mediated defense signaling (Studham and MacIntosh, 2013). Taken together, these results point toward a complex role of phytohormones in plant–pathogen and plant–herbivore interactions under drought stress.

Besides changes to plant signaling pathways, drought stress alters nutritional quality of the phloem (Huberty and Denno, 2004). Plants produce nitrogen-related osmoprotectants to counter the low osmotic pressure that occurs in response to drought stress resulting in increased nitrogen content in phloem sap (Brodbeck and Strong, 1987). Increased levels of proteins and amino acids have also been reported in leaf tissue during drought stress which may minimize water loss (Garg et al., 2001; Johnson et al., 2011). Analysis of aphid feeding behavior indicates that drought stress increases mesophyll/phloem resistance (Guo et al., 2016) plausibly due to change in phloem sap viscosity due to altered sugar and solute concentrations increasing the difficulty for aphids to acquire nutrients (Hsiao, 1973; Garg et al., 2001). Changes in the water potential of the host plant due to water stress can also impact the aphids’ ability to consume xylem sap which allows aphids to deal with the high sugar concentration and osmotic pressure of the phloem sap (Pompon et al., 2010, 2011; Guo et al., 2016). Therefore, an integrative approach evaluating changes in gene expression and analyzing changes in host plant quality is essential to develop a better understanding of the impacts of water stress on plant–aphid interactions.

In the present study, we sought to investigate factors that influence performance of soybean aphids (*Aphis glycines* L.) and aphid-transmitted *Soybean mosaic virus* (SMV) on water-stressed soybean (*Glycine max* L.) plants. We adopted a broad approach by investigating plant nutritional quality and defense signaling as possible mechanisms underlining the interaction between water stress, herbivory and virus transmission. Our experimental setup comprised of three water stress regimes: drought, well-watered and saturated and two levels of aphid infestation: viruliferous (SMV-infected) and non-viruliferous (uninfected). We analyzed soybean aphid population growth, feeding behavior using electrical penetration graph (EPG) technique, and monitored virus infection and transmission. Further, we measured total and individual amino acid profiles and gene expression related to major plant signaling pathways in plants subjected to water stress, insect feeding and virus infection.

## Materials and Methods

### Plant Growth Conditions

Soybean variety Asgrow^®^ AG3432 (Monsanto, St. Louis, MO, USA) was grown in Mastermix^®^ 830 soilless media (Mastermix, Quakertown, PA, USA). All plants were maintained at 60–70% relative humidity, temperature of 24 ± 1°C and a photoperiod of 16:8 (L:D) hours (h) at a photosynthetically active radiation (PAR) of 460 μmol/m^2^/sec in an environmental chamber. Plants were watered three times per week ad libitum and received Miracle Gro^®^ (Scott’s Company, Marysville, OH, USA) solution as per label instructions once per week.

### Virus Source

Soybean mosaic virus-infected seeds were provided by Dr. Glen L. Hartman, Laboratory for Soybean Disease Research at the University of Illinois, Urbana–Champaign. SMV was maintained through both mechanical inoculation and aphid transmission (Hunst and Tolin, 1982; Hill et al., 2001). Young leaves from SMV-infected plants were ground in 0.01 M phosphate buffer (pH 7.1). Virus inoculum was rub-inoculated using a cotton-tipped applicator to carborundum-dusted leaves. At least 2–3 almost fully expanded leaves were inoculated per plant. After 5 min leaves were then gently rinsed with water to remove excess carborundum. SMV infection was monitored and confirmed through presence of symptoms and RT-PCR analysis using primers listed in **Table 1**.

**Table 1:** Quantitative reverse transcription -PCR (qRT-PCR) primer pair sequences and corresponding PCR efficiencies.

Gene	Locus/description	Primer sequences	PCR efficiency	Amplicon length (bp)	Reference
*Internal Control*					
*FBOX*	Glyma.12g051100/F-box only protein	AGATAGGGAAATTGTGCAGGT	2.05	93	Le et al., 2012
		CTAATGGCAATTGCAGCTCTC			
*SA marker genes*					
*PR1*	Glyma.15g06790/Pathogenesis related protein 1	GCAGCTAGCAAGCTACCACT	2.26	196	Li et al., 2008
		CACGCCACAACGTTCAAGA			
*PAL2*	Glyma.20g32135.1/Phenylalanine ammonia lyase	TCAGAAGCAAATGCTGCCAAC	1.88	144	This paper
		CTCTAGCATGCGCTTGACCT			
*JA marker genes*					
*JAR1*	Glyma.16g03010.1/Jasmonic acid-amido synthetase	ACACCAAGATTCTCCTAGCTGC	1.75	208	This paper
		AGGATCCGTCCTCCCATTCA			
*AOS*	Glyma.17g36530/Allene oxide synthase	TCCTCAACCAAACAACGCTCT	1.98	210	Studham and MacIntosh, 2013
		GCGGGACTTGAAGAACTCGT			
*ABA marker genes*					
*RD20A*	Glyma03g41030/Responsive to desiccation 20	GTGGCACATGACTGAAGGAA	1.98	195	Neves-Borges et al., 2012
		ATCTTTCCAGCAGCACCTCT			
*SCOF1*	Glyma.17g35430/Soybean zinc finger protein	GAGGTAAGGCCCATGAGTGC	1.86	224	Studham and MacIntosh, 2013
		CGAAAAATCCGGAAAGGCCG			
*Virus marker*					
*SMV413-CP*	GU015011/ Soybean mosaic virus coat protein	TTCCAATGGTTGAAGGAAG	1.93	456	This paper
		CTTGCCCTGTTTGGTGTTTT			

### Insect Source

Soybean aphids were originally collected from a soybean field at the Pinney Purdue Agricultural Center (PPAC), Watanah, Indiana. In the laboratory, the aphid colony was maintained on AG3432 at temperature of 24 ± 1°C and a photoperiod of 16:8 (L:D) h in 30 × 30 × 76 cm insect cage (BioQuip, Rancho Dominguez, CA, USA). In order to obtain viruliferous aphids, adult non-viruliferous aphids from the lab colony were exposed to SMV-infected plant for 30 min (Clark and Perry, 2002).

### Water-stress Treatments

To determine the level of water stress to be applied, a modified water stress procedure was used (Porcel and Ruiz-Lozano, 2004). Briefly, 500 g of Mastermix 830 soilless media (Mastermix, Quakertown, PA, USA) was weighed, and fully saturated with water in 6′′ pots (Hummert International, Earth City, MO, USA). Saturated media was weighed and a Waterscout^®^ SM100 soil moisture probe (Spectrum Technologies, Aurora, IL, USA) was used to determine percent volumetric water content (VWC). The saturated media was allowed to air dry until all moisture was lost. VWC and weight of water lost were monitored daily. A calibration curve based on average VWC and corresponding mass of water was computed based on which the volume of water to be used to maintain each water stress treatment was determined (Supplementary Figure S1). Upon reaching V1 or first trifoliate leaf stage, plants were exposed to three water-stress treatments: drought-stress conditions (25% field capacity or FC corresponding to 7.6% VWC), well-watered conditions (75% FC corresponding to 17.9% VWC), and water-saturated or flooding condition (100% FC corresponding to 24.8% VWC). The plants were maintained at such conditions for 3 days prior to the start of the experiment and for 7 days during the duration of the experiment. In order to maintain the water stress conditions, soil water content of the soilless media was measured daily (early evening) and re-watered to restore the soil water content to required levels. During the 24 h period between measurement and re-watering, the water content only decreased by about 2–6% VWC in each of the treatments. Leaf water potential measurements were not performed because the protocol used has been previously shown to reduce water potential in drought stressed plants (Porcel and Ruiz-Lozano, 2004). With respect to the saturation treatment, previous studies have found that flooding or soil water saturation does not impact leaf water potential (Oosterhuis et al., 1990).

### Experimental Design and Structure

The experimental design was a 3 × 3 factorial with three water stress treatment levels (drought, well-watered, and saturated) and three aphid infestations (uninfested plants, plants infested with non-viruliferous aphids, and plants infested with viruliferous aphids). For all treatments, 20 adult aphids were transferred using a camel-hair brush onto the adaxial surface of the first true leaves (V1 growth stage).

To assess quality of water-stressed plants as a food resource, aphid fecundity was measured daily by counting the number of nymphs and adults until day 7. At the end of the experiment, fresh weight and dry weight of the plants were obtained in order to compute water content and biomass in plants. Each treatment was replicated 3–6 times and the experiment was repeated three times (biological replicates).

### Absolute Quantification of SMV

In order to accurately determine virus level, the SMV-coat protein gene was quantified from infected leaf tissue using SMV413-CP primers whose sequence are listed in **Table 1**. The PCR program consisted of 95°C for 5 min denaturation stage followed by 40 cycles of 95°C for 1 min, 55°C for 30 s, 72°C 30 s elongation, and a final elongation step of 5 min at 72°C. The PCR product was cloned following manufacturer’s protocol (pCR8/GW/TOPO^®^ vector, Thermo Scientific, Pittsburgh, PA, USA) and sequenced. The nucleotide sequences were 100% identical to the target sequences deposited in GenBank (Accession: GU015011). To quantify SMV level in a given leaf tissue, a standard curve was prepared using the aforementioned plasmid containing the SMV-coat protein target region at a known concentration of 187.8 ng/μL. The mass of the plasmid containing insert was estimated from the size of the plasmid 3,273 base pairs and the average molecular mass of a base pair in DNA 1.096 × 10^-21^ g, resulting in the mass of one copy of the plasmid being equal to 3.59 × 10^-18^ g. The initial concentration of the plasmid standard was adjusted in water to be 1.0 × 10^10^ copies/μL. To obtain a standard curve, 10-fold serial dilutions (ranging from 1 × 10^7^copies/μL to 1 × 10^1^copies/μL) from the initial concentration of plasmid. Quantitative polymerase chain reaction (qPCR) was then performed on plant tissue samples. Reactions for serial dilutions were performed in triplicates.

### Electrical Penetration Graph

Aphid feeding behavior was analyzed using the electrical penetration graph technique (EPG) on a GIGA 8 complete system (EPG Systems, Wageningen, Netherlands) (Tjallingii and Esch, 1993). Adult soybean aphids were starved for 1 h prior to wiring. After wiring of aphids was completed, eight plants, two per water stress treatment were placed into a Faraday cage. Treatments were both tested and analyzed blindly. The wired plant electrodes were then placed into the soil, and insect probes adjusted to that the aphids could rest on the underside of the first trifoliate leaf allowing for contact between the plant and insect. Aphids were then allowed to feed for 8 h, while the aphid feeding behavior was recorded. This experiment was repeated until sufficient biological replications were obtained. Each feeding experiment was analyzed to determine the amount of time spent in each of the four main phases: pathway phase (PP), non-probing phase (NP), sieve element phase (SEP), and xylem phase (XP). Other parameters that were recorded include time to 1st probe, time to first potential drop (PD) and the number of PDs all of which provide an indication of aphid health/condition and also virus acquisition and transmission (Martin et al., 1997). EPG results were analyzed using Stylet+ software (EPG Systems, Wageningen, Netherlands).

### Petiole Exudate Collection

In a separate experiment, plants were grown in pots with a 12′′ diameter (Myers Industries Marysville, OH, USA) until the V1 growth stage. Plants were then subjected to moisture stress as described above and phloem exudates were extracted as per Nalam et al. (2012). In order to prevent bacterial contamination of the petiole exudates, trifoliates were cut and immersed in 50% ethanol, and then immediately moved to 0.05% bleach solution for no more than 2–3 s in to achieve surface sterilization of the leaf and cut surfaces. Trifoliates were then transferred to 1 mM EDTA solution (pH 8.0) until all of the trifoliates were collected from the treatment groups. Next, 1 cm of the stem was cut off, and three trifoliates were immediately placed into wells containing 4 mL of 1 mM EDTA. After all the trifoliates were transferred to fresh EDTA buffer, they were placed in an aquarium with a clear lid lined with moistened paper towels for 24 h. Petiole exudates from three wells were then pooled per sample resulting in nine trifoliates per pooled sample. Samples were then filtered through 0.2 μm pore size filters and lyophilized. After lyophilization, samples were eluted in 750 μL of 1 mM EDTA solution and used in artificial feeding assays and nutrient analysis (described below).

### Artificial Feeding Assay

An artificial diet previously tested for optimum soybean aphid performance (Diet C, Wille and Hartman, 2008), was used for all artificial feeding assays. An artificial feeding chamber consisted of 55 mm petri dishes (VWR, Radnor, PA, USA) with parafilm (Bemis, Neenah, WI, USA) stretched across the top to act as feeding sachets. Each sachet contained a total volume of 750 μL, which included the artificial diet with or without 25 μL petiole exudates or buffer used to collect the petiole exudates. Ten 3rd instar non-viruliferous aphid nymphs were placed in each feeding chamber and allowed to develop until adulthood. Total number of nymphs and adults were counted at the end of the experiment. We did not test viruliferous aphids because SMV is not a phloem-limited virus and thereby not likely to affect aphid biology in artificial feeding assays.

### Amino Acid Analysis

Petiole exudates from each of the water-stress treatments were sent to the Donald Danforth Plant Science Center (St. Louis, MO, USA) for amino acid analysis. Samples were tested for amino acids using the AccQTag derivitization method (Waters Corporation, Milford, MA, USA). Samples were run in triplicate on an Acuity UPLC^®^ System for 9.5 min and essential and non-essential amino acids were detected. Results from amino acid analysis were standardized to the average leaf mass for each treatment.

### RNA Extraction, cDNA Synthesis and Reverse Transcriptase-Quantitative PCR (RT-qPCR)

For all experiments, 100 mg leaf tissue was harvested from each of the nine treatments (3 water stress × 3 aphid infestations), flash frozen, and stored at -80°C for further processing. Plant RNA was extracted using the Trizol^®^ (Invitrogen, Grand Island, NY, USA) method, checked for purity and quantity using a Nanodrop ND 100 (Thermo Scientific, Pittsburgh, PA, USA). RNA was then treated with Turbo DNase^®^ (Invitrogen, Grand Island, NY, USA) in order to remove DNA contamination. Complete removal of DNA was verified by PCR using DNase treated RNA as template for amplification with the internal control *FBOX* gene (Le et al., 2012). Two micrograms of RNA was used as a template for cDNA synthesis using the Verso^®^ cDNA synthesis kit (Thermo Scientific, Pittsburgh, PA, USA).

RT-qPCR was performed using SYBR Green^®^ (Biorad, Berkeley, CA, USA) on a CFX Connect^®^ (Biorad, Berkeley, CA, USA) thermocycler. The cycling conditions used were: 95°C for 2 min, followed by 40 cycles of 95°C for 30 s, and 60°C for 30 s. PCR efficiencies (E) of target and internal control genes were determined using the LinRegPCR software (Ruijter et al., 2009) and are shown in **Table 1**. Reactions for all samples were performed in duplicate and three biological replicates and a negative and positive control were used in each run. Fold change was determined by normalizing transcript levels of the genes of interest to the internal control gene (*FBOX*), followed by normalization to expression of the respective gene in a plant that was not subjected to water stress or aphid infestation using the following formula, 2^-ΔΔCT^ (Livak and Schmittgen, 2001). Fold changes were log_2_ transformed in order to normalize data. Log_2_ (fold change) data is presented and also used for all statistical analysis.

### Statistical Analysis

All response variables conformed to assumptions of ANOVA and no transformations were performed with the exception of gene expression fold change. To determine the relationship between water content of plants and soil VWC a simple linear regression analysis was performed and 95% confidence intervals were calculated. Regression analysis was performed in Sigma Plot Version 12.5 (Systat Software^®^ San Jose, CA, USA). To determine if water-stress treatments affected aphid population growth rate, a two-way ANOVA was conducted with water stress (drought, well-watered, saturated) aphid infestations (non-viruliferous and viruliferous) and their interactions as main effects. Fold change of plant defense genes was also analyzed using two-way ANOVA with the same fixed and interaction effects. To determine the effect of petiole exudates (from water-stressed plants) on aphid populations in artificial feeding assays, a one-way ANOVA was performed. A one-way ANOVA was also conducted to determine the effect of water stress on virus level (log copies) and amino acid levels. For EPG analysis, the mean time spent by the aphids in various activities was analyzed using non-parametric Kruskal–Wallis test. Parameters that showed a significance level close to 5% were further analyzed using a separate pair-wise comparison (Mann–Whitney *U*-test, α = 0.05). All data was analyzed using Minitab Version 17 (Minitab^®^ State College, PA, USA).

## Results

### Effect of Water Stress on Plant Growth and Water Content

**FIGURE 1:**
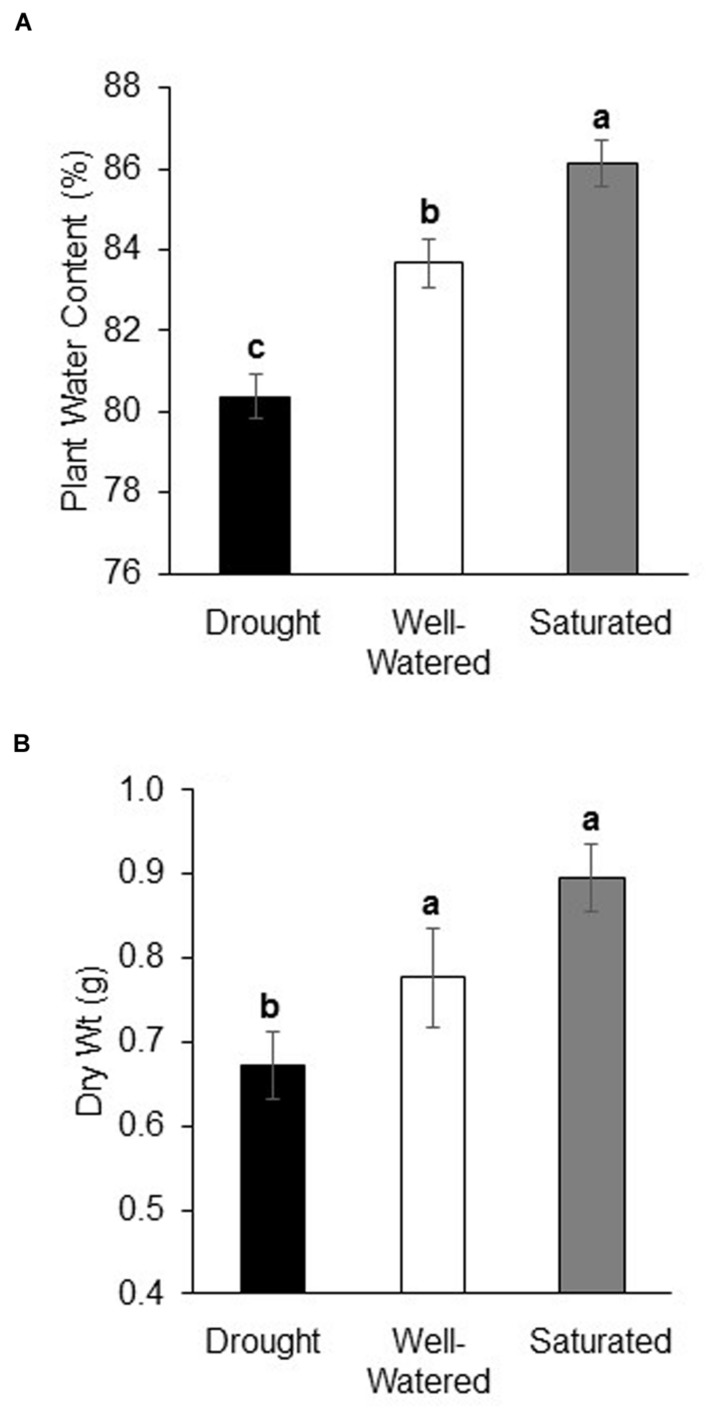
**Drought stress reduces plant water content and dry weight. (A)** Water content (%) and **(B)** dry weight (g) of soybean plants subjected to drought and saturation treatments for a 10 days period. Soybean plants grown under well-watered conditions serve as control. Each bar represents the mean ± SE of *n* = 3–6 plants per experiment or biological replicate. Each experiment was repeated three times. Different letters indicate significant difference between treatments (Tukey’s HSD *P* < 0.001).

Water content was lowest in plants under drought and highest in plants under saturation (*P* < 0.001, **Figure 1A**). At the end of the experiment, there was a strong positive relationship between soil and plant water content indicating that water-stress treatments were consistent throughout experiments (*R*^2^: 0.92 uninfested plants, 0.82 non-viruliferous aphid-infested plants, 0.84 viruliferous aphid-infested plants). Plant dry weight measured at the end of the experiment also showed the same pattern (*P* < 0.001) except there was no difference in dry weight between well-watered and saturated plants (**Figure 1B**). The expression of a drought-stress marker, *RD20A* (Neves-Borges et al., 2012), 3 days after the commencement of water stress, treatments showed a significant increase in expression under drought stress as compared to well-watered and saturated plants (Supplementary Figure S2). Feeding by either non-viruliferous or viruliferous aphids did not affect plant water content or dry weight in response to the water-stress treatments (Data not shown).

### Effect of Water Stress on Aphid Populations

**FIGURE 2:**
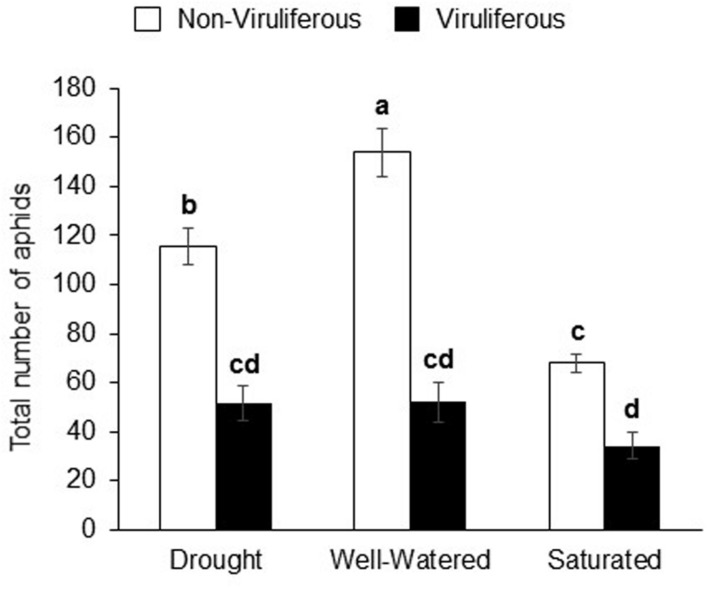
**Water stress and virus infection has a significant impact on aphid populations on soybean plants.** The total number of aphids (Adults + nymphs) on soybean plants 7 days post-infestation. Each bar represents the mean ± SE of *n* = 3–6 plants per experiment or biological replicate. Each experiment was repeated three times. Different letters indicate significant difference between treatments (Tukey’s HSD *P* < 0.001).

Water-stress treatments had significant and strong effects on aphid populations. The interaction effect (water stress × aphid infection levels) and main effects were significant for aphid populations (Supplementary Table S1). Non-viruliferous aphid populations were highest on well-watered plants and lowest on saturated plants (**Figure 2**). On the other hand, there was no significant difference in viruliferous aphid populations on any of the water-stress treatments (**Figure 2**). However, populations of viruliferous aphids were significantly lower than non-viruliferous aphids under all treatments.

### Effect of Water Stress on SMV Infection and Transmission

**FIGURE 3:**
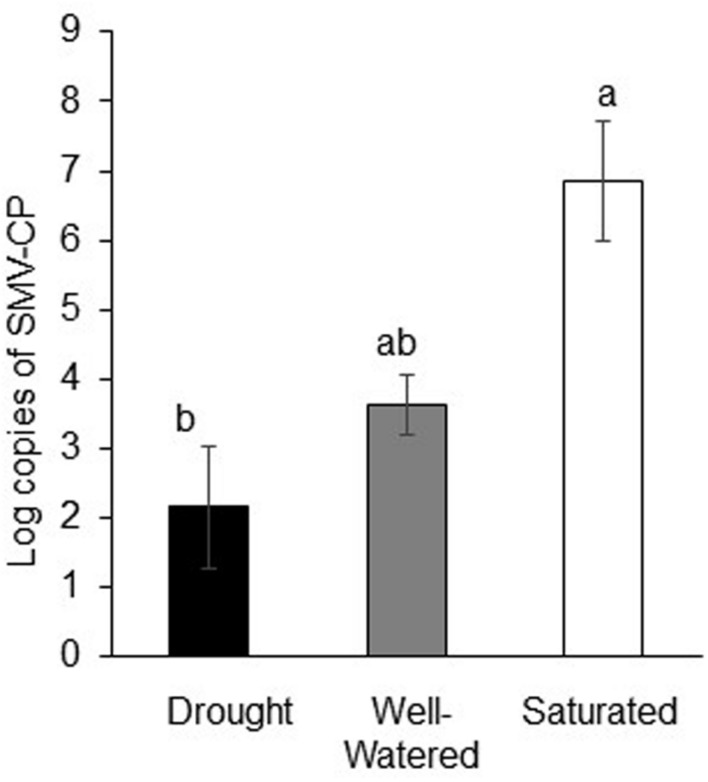
**Drought reduces virus infection but saturation enhances virus infection.** Log copies of SMV-coat protein in soybean plants subjected to water stress and feeding by viruliferous aphids. Each bar represents the average of *n* = 3–6 plants per experiment or biological replicate. Each experiment was repeated three times. Different letters indicate significant difference between treatments (Tukey’s HSD *P* < 0.001).

Water-stress treatments also significantly impacted virus infection levels and aphid’s ability to transmit SMV. Although there was no significant difference in viruliferous aphid populations under the different water-stress treatments (**Figure 2**), virus levels as measured by average number of SMV-coat protein molecules per 100 mg of leaf tissue differed between treatments. SMV infection was highest in saturated plants and lowest in drought-stressed plants (*P <* 0.001; **Figure 3**). Transmission rate (calculated as the proportion of soybean plants testing positive for the virus) showed similar patterns in that rates were lowest under drought stress and highest under saturated conditions, 50 and 77%, respectively, compared to well-watered plants where the rate was 60%

### Aphid Feeding Behavior on Water Stressed Plants

**FIGURE 4:**
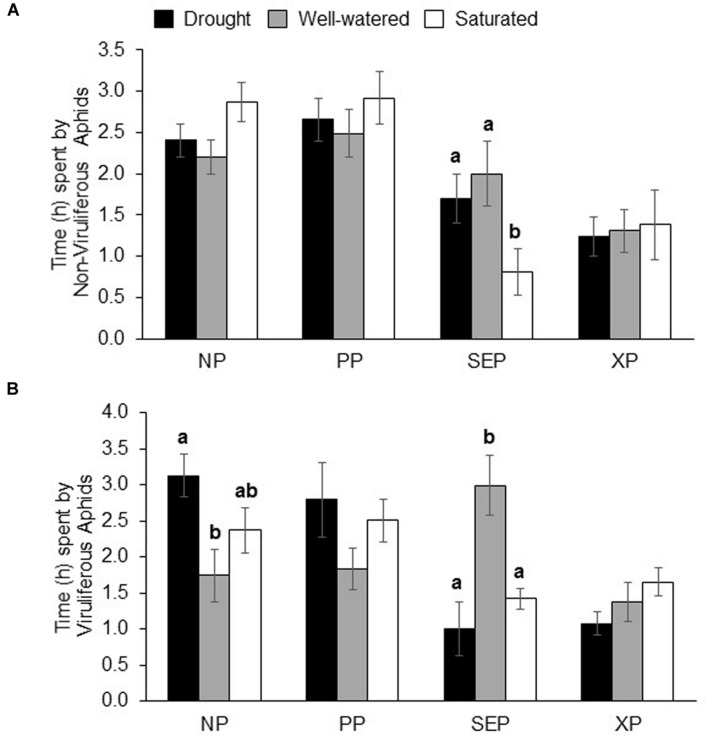
**Electrical penetration graph analysis of aphid behavior on water-stressed plants.** Time (in h) spent by **(A)** non-viruliferous and **(B)** viruliferous aphids on drought, well-watered and saturated soybean plants over an 8 h of recording time. Each values represents the mean from 12 to 19 replications. The time spent by aphids on various activities (NP, non-probing phase; PP, pathway phase; SEP, Sieve element Phase; XP, Xylem Phase) was analyzed by the non-parametric Kruskal–Wallis test (*P* < 0.05). For parameters that showed a significance level of *P* ≤ 0.05 a separate pairwise comparison using the Mann–Whitney *U*-test (α = 0.05) was performed. Each bar represent the mean ± SE. Different letters indicate significant difference between treatments.

Among the feeding behaviors recorded, a significant impact of water stress was observed on the amount of time spent by non-viruliferous aphids in the sieve-element phase, SEP (*P* = 0.02; **Figure 4A**). Non-viruliferous aphids spent significantly less time in the SEP on saturated plants compared to drought and well-watered plants (**Figure 4A**). There were significant differences in the time spent by viruliferous aphids in both SEP and non-probing phase, NP (*P* = 0.02 and *P* = 0.05, respectively; **Figure 4B**). Viruliferous aphids spent lesser time in SEP on both drought-stressed and saturated plants compared to well-watered plants (**Figure 4B**). Viruliferous aphids spent more time in NP on plants under drought and saturation treatments compared to on plants that were well-watered plants (**Figure 4B**). Water-stress treatments did not affect aphid hydration status (Supplementary Figure S3).

Irrespective of the water-stress treatment, non-viruliferous aphids showed a significantly greater number of (PDs; i.e., when the stylet tip punctures a cell) compared to viruliferous aphids (**Table 2**). Additionally, non-viruliferous aphids took significantly less time to 1st PD under all conditions. In terms of behaviors critical for virus transmission, viruliferous aphids took least amount time for 1st PD under saturated conditions followed by well-watered and drought stress treatment (**Table 2**).

**Table 2:** Probing behavior of non-viruliferous and viruliferous aphids on drought-stressed, well-watered and water-saturated plants.

Parameter		Drought		Well-Watered		Saturated		
		Non-viruliferous	Viruliferous	Non-viruliferous	Viruliferous	Non-viruliferous	Viruliferous	
	*n*	*19*	*16*	*16*	*14*	*13*	*12*	*P*-value
Number of PD	(#)	81.4 ± 5.3 a	45.6 ± 3.5 b	78.8 ± 6.9 a	51.1 ± 5.8 bc	70.9 ± 6.9 ac	59.0 ± 6.0 bc	0.001
Time to 1st potential drop (PD)	(min)	0.7 ± 0.1 a	5.0 ± 0.2 b	0.8 ± 0.2 a	3.0 ± 1.0 b	0.8 ± 0.3 a	1.4 ± 0.5 a	<0.0001
Time to 1st probe	(min)	42.5 ± 7.9 a	91.8 ± 14.4 b	47.7 ± 10.4 a	111.4 ± 20.8 b	36.6 ± 10.9 a	109.7 ± 15.6 b	<0.0001
Aphids with phloem phase	(#), (%)	17, 89.5	12, 75	16,100	14, 100	9, 69.2	12, 100	
Aphids with xylem phase	(#), (%)	17, 89.5	16, 100	14, 87.5	14,100	10,76.9	11, 91.7	

*Total durations (min), numbers (#), or percentage of total time (%). Means followed by different letters within lines are significantly different.*

### Analysis of Petiole Exudates from Water Stressed Plants on Aphid Populations

A total of 18 amino acids were detected including both essential and non-essential amino acids (**Table 3**). There were significant differences in total amino acid content in vascular sap enriched petiole-exudates from water-stressed plants (*P <* 0.001). Petiole-exudates from drought-stressed plants had greater total amino acid content, but not significantly different from well-watered plants. Petiole-exudates from saturated plants had the lowest free amino acid content (**Table 3**). There were significant differences in eight amino acids due to water stress including four essential amino acids: isoleucine, leucine, threonine, and valine and four non-essential amino acids: asparagine, glutamic acid, proline (marginally significant), and tyrosine (**Table 3**). All amino acids were higher in petiole-exudates from drought-stressed plants compared to well-watered and saturated plants with the exception of tyrosine (**Table 3**).

**Table 3:** Concentrations of amino acid in petiole exudates of soybean plants subjected to different water-stress treatments.

Amino Acids	*P-values*	Drought	Well-Watered	Saturated
Alanine	0.302	182.74	119.92	17.81
Arginine	0.569	167.16	114.30	21.52
**Asparagine**	**0.05**	**23322.58 a**	**13481.85 ab**	**6316.14 b**
Aspartic Acid	0.376	2621.01	2262.47	1660.27
Glutamine	0.085	5735.13	5425.74	1788.18
Glycine	0.513	745.11	744.96	156.32
**Glutamic Acid**	**0.01**	**1229.81 a**	**536.74 b**	**507.06 b**
**Isoleucine**	**<0.001**	**528.75 a**	**214.88 b**	**27.83 c**
**Leucine**	**0.015**	**304.09 a**	**138.77 ab**	**48.64 b**
Lysine	0.467	218.89	277.51	29.95
Methionine	0.086	37.75	16.99	6.52
Phenylalanine	0.214	293.58	236.13	54.27
Proline	0.065	409.66	94.78	10.51
Serine	0.155	1490.07	2298.38	237.59
**Threonine**	**0.007**	**832.11 a**	**317.36 b**	**197.26 b**
Tryptophan	0.298	51.67	66.17	20.91
**Tyrosine**	**0.04**	**124.04 ab**	**159.50 a**	**25.23 b**
**Valine**	**0.005**	**555.48 a**	**261.68 ab**	**88.76 b**
**Total**	**<0.001**	**43445.36 a**	**37500.14 ab**	**12347 b**

*Values are expressed as pmol/gm fresh weight. Values represent mean of *n* = 5 plants or replicates. Means followed by different letters indicate significant difference between treatments. Treatments with significant differences are in bold.*

**FIGURE 5:**
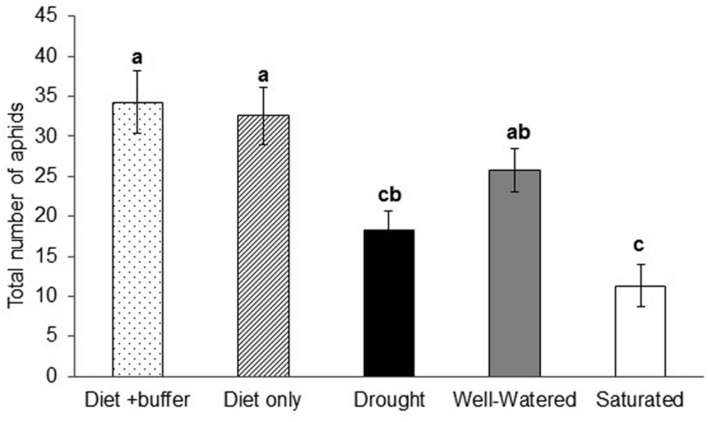
**Petiole exudates from water stressed plants alters aphid populations.** Total number of aphids reared on artificial diet supplemented with buffer or petiole exudates collected from drought, well-watered and saturated plants in artificial feeding assays. Each bar represents the mean ± SE of *n* = 8 artificial feeding assays or replicates. Different letters indicate significant difference between treatments (Tukey’s HSD *P* < 0.001).

Artificial feeding assays performed using petiole-exudates from soybean plants exposed to the various water-stress treatments indicated that aphid populations were highest on artificial diet and diet plus buffer, which served as the positive controls (*P* < 0.0001; **Figure 5**). Among the water-stress treatments, soybean aphid populations were highest in response to petiole-exudates from well-watered plants, which was not significantly different from the positive controls (**Figure 5**). The lowest aphid populations were observed in response to petiole-exudates collected from saturated plants which was not significantly different from drought stressed plants. Overall, non-viruliferous aphid numbers in artificial feeding assays showed the same pattern as aphid populations on whole plants (**Figure 2**).

### Gene Expression Analysis

**FIGURE 6:**
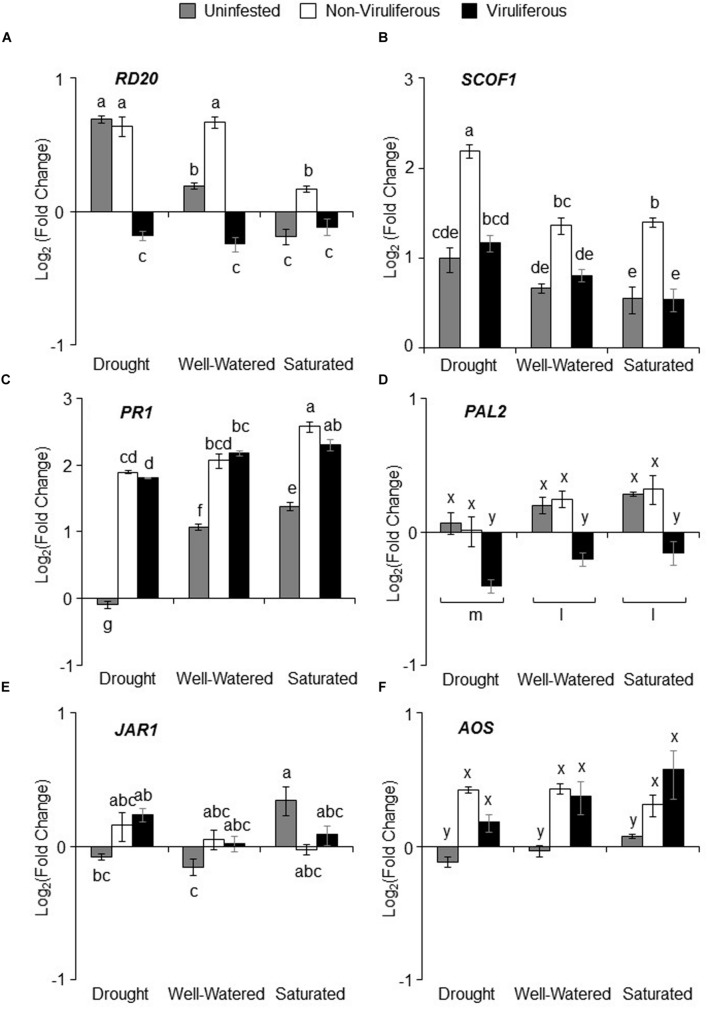
**Water stress and aphid feeding affects plant defense signaling genes.** Log_2_ (fold change) with respect to uninfested unstressed control of the following genes **(A)** *RD20A* (ABA/Drought marker), **(B)** *SCOF1* (ABA marker), **(C)** *PR1* (SA marker), **(D)** *PAL2* (SA biosynthesis), **(E)** *JAR1* (JA marker) and **(F)** *AOS* (JA biosynthesis). Relative gene expression and fold change was calculated using the comparative 2^-ΔΔC^_T_ method with *FBOX* as endogenous control. Values are shown as mean of Log_2_ (fold change) ± SE. Each bar represents the average Cq values derived from of *n* = 3–6 plants pooled together from three independent experiments. Different letters indicate significant difference between treatments (Tukey’s HSD *P* < 0.001).

There was significant interaction between water stress and aphid infestation on ABA marker genes, *RD20A* but not *SCOF1* (Supplementary Table S2). *RD20A* has been previously shown to be highly induced by drought stress, and indeed we found a significant increase in *RD20A* expression in response to drought stress (**Figure 6A**). In contrast, saturation resulted in the suppression of *RD20A* expression (**Figure 6A**). Feeding by non-viruliferous aphids increased *RD20A* expression under all treatments (**Figure 6A**). There was a moderate increase in *SCOF1* expression under drought stress compared to well-watered and saturated conditions albeit not statistically significant. Similar to *RD20A*, non-viruliferous aphid feeding up-regulated *SCOF1* expression under drought stress (**Figure 6B**). The SA pathway marker, *PR1* was affected by the interaction between water stress and aphid infestation but not *PAL2*, a gene involved in SA biosynthesis (Supplementary Table S2). *PR1* expression was down-regulated in uninfested drought-stressed plants and significantly up-regulated in saturated plants. In addition, *PR1* expression was induced in response to both non-viruliferous and viruliferous aphid feeding in all treatments (**Figure 6C**). *PAL2* expression was reduced under drought stress, moreover, expression was down-regulated in response to feeding by viruliferous aphids (**Figure 6D**). Expression of the JA marker, *JAR1* was also affected by the interaction between water stress and aphid infestation, but only water stress had a significant main effect (Supplementary Table S2). *JAR1* expression was suppressed in drought-stressed plants and in well-watered plants whereas, expression was significantly up-regulated in saturated plants (**Figure 6E**). The expression of *AOS*, involved in JA biosynthesis, was not affected by water stress (Supplementary Table S2). However, the pattern of expression was similar to *JAR1*. In general, feeding by either non-viruliferous or viruliferous aphids significantly up-regulated *AOS* expression as compared to the uninfested control (**Figure 6F**).

## Discussion

We investigated plant responses to simultaneous exposure to abiotic (drought and saturation) and biotic stresses (insect feeding and virus transmission) in the model crop species, soybean at two levels: organismal (measured as fecundity, feeding behavior, and virus transmission), and sub-organismal (measured as free amino acid profiles and defense gene expression). Our results show that drought and saturation have different consequences for plant resistance to aphids and aphid-transmitted SMV. We hypothesize that these outcomes are a result of changes in amino acid content and interactions between the phytohormones ABA, SA, and JA.

Availability of water whether excess or deficit is critical for plant growth and maintenance, which are important determinants for plant resistance against insect herbivores and pathogens. In the current study, soybean plants were subjected to drought and saturation as per Porcel and Ruiz-Lozano (2004). Similar methods of water-stress treatments have been used in other studies that have attempted to elucidate the impact of drought stress on phloem-feeding insects (Mewis et al., 2005, 2012; Khan et al., 2010; Guo et al., 2016). The water stress regime implemented in the current study correlated well with plant water content at the end of the experiment; water content was lowest in plants under drought and highest in plants under saturation. Moreover, there was a strong positive relationship between soil and plant water content indicating that water-stress treatments were consistent throughout experiments. The increase in plant water content in saturated plants may suggest that they did not experience significant stress as a result of the treatment. A previous study did not find any impact of saturation on plant water content at day 7 but found that the total N content of the plants was significantly reduced (Bacanamwo and Purcell, 1999). So, it is possible that although a change in plant water content was not observed, the treatment significantly affected plant metabolism. Daily variation in VWC was minimized by maintaining the plants in an environmental chamber that was maintained at a constant temperature and humidity. Furthermore, daily monitoring of the VWC revealed that over a 24 h between re-watering, the VWC reduced by only 2–6% depending on the treatment with the highest fluctuation observed under saturated conditions. The effectiveness of drought treatment was further confirmed by the strong induction of *RD20A*, a known soybean drought-stress marker in drought stressed plants after 3 days of treatment. Taken together, these results confirm the reliability of water-stress treatments imposed in the current study.

Water stress especially drought in host plants is known to impact aphid performance (Huberty and Denno, 2004). We show that populations of non-viruliferous aphids were significantly reduced when plants were saturated or drought-stressed compared to well-watered conditions. This effect was reflected in aphid feeding behaviors as determined by EPG analysis. We found that non-viruliferous aphids spent the least amount of time feeding from the sieve element (SEP) on saturated plants, which likely affected growth of aphid populations. In contrast, aphids tended to spend the greatest amount of time consuming sap from the phloem of well-watered plants and their populations grew significantly faster. Previously, it has been reported that drought stress resulted in increased populations of a generalist aphid, *M. persicae*, on broccoli or *Arabidopsis* plants, whereas saturation negatively impacted population growth (Khan et al., 2010; Mewis et al., 2012). In contrast, drought and saturation had no impact on the specialist, *Brevicoryne brassicae*. The authors found that drought and saturation increased secondary metabolite levels, which the specialist, *B. brassicae* were better able to tolerate compared to generalist, *M. persicae*. In the current study, however, populations of the specialist, soybean aphid, were reduced under drought stress, and saturation. There could be several reasons for the variation in outcomes from one system to another including plant hosts used (Hale et al., 2003), insect species, severity and type of stress (Huberty and Denno, 2004; Mody et al., 2009), and even experimental design (Koricheva et al., 1998). To summarize, soybean aphid performance was best on well-watered soybean plants owing to longer undisturbed feeding from the sieve element compared to saturated and drought-stressed plants where feeding from SEP was reduced.

Virus infection has been shown to improve drought tolerance in a variety of crop species (Xu et al., 2008). For instance, it was recently demonstrated that BYDV-infected wheat plants had increased growth, seed set, and germination compared to non-infected plants under drought stress (Davis et al., 2015b). In addition, fecundity of the aphid vector, *R. padi* increased by 47% when fed on BYDV-infected drought-stressed plants whereas fecundity increased by only 23% from feeding on BYDV-infected well-watered plants. Unlike the abovementioned study where plants were first inoculated with the virus, we first subjected plants to water stress and then exposed them to viruliferous aphids. By introducing aphids after application of water treatments we were not only able to monitor aphid performance but also virus transmission on water-stressed plants. Viruliferous aphid population was significantly reduced compared to non-viruliferous aphids irrespective of the water treatment. Our findings corroborate a previous report that showed that SMV infection negatively affects population growth of soybean aphids on soybeans (Donaldson and Gratton, 2007). Yet, we found that drought-stressed plants harbored lowest SMV infection and had the lowest transmission rate whereas saturated plants had highest level of infection and transmission rate. It has been previously shown that the time taken to first intracellular puncture or PD and the number of PD are important for efficiency of virus transmission in case of non-persistently transmitted plant viruses (Martin et al., 1997). Virus infection and transmission rate observed in the current study correlated well with aphid feeding behaviors on the respective plants. Viruliferous aphids took a longer time to first PD on drought-stressed plants and shortest time on saturated plants. Moreover, the number of PD was lowest on drought-stress plants and highest on saturated plants albeit not statistically significant. This response correlated well with the lower SMV level observed in plants under drought stress and highest under saturation. This is in contrast to a previous report that showed that drought stress increased aphid movement resulting in increased BYDV transmission (Smyrnioudis et al., 2000). The outcomes of the interaction between water stress and insect-transmitted disease is dependent on several abiotic and biotic factors (Bartels and Sunkar, 2005; Davis et al., 2015b). It is possible that mode of virus transmission, persistent (pathogen propagates within the vector) such as BYDV or non-persistent (pathogen does not propagates within the vector) such as SMV influences the outcomes. In non-persistent or stylet-borne virus, virions are attached to the distal tip of the stylet of the insect and when the insect feeds on a healthy plant, it inoculates the plant with the virus. In this case, transmission efficiency is greatest when vectors briefly puncture plant cells and decreases with longer feeding. In contrast, transmission efficiency increases with longer feeding duration in case of persistently transmitted viruses (Purcell and Almeida, 2005). It is plausible that enhanced host plant traits and vector performance is critical for transmission of persistent viruses compared to non-persistent viruses.

Water stress can modify nutritional quality of the phloem sap which has significant repercussions for aphids (Huberty and Denno, 2004). We therefore collected vascular sap-enriched petiole exudates from soybean plants exposed to the various water-stress treatments for artificial feeding assays. The collection of petiole exudates in the current study was modified from the protocol developed by King and Zeevaart (1974), which makes use of a chelating agent EDTA to enhance exudation from the cut petioles. Although criticized for the use EDTA that can hinder the identification of free amino acids this method has been used in several studies (Urquhart and Joy, 1981; Douglas, 1993; Karley et al., 2002; Khan et al., 2010; Mewis et al., 2012; Nalam et al., 2012; Guo et al., 2013; Zhang et al., 2015). We believe that the low concertation of EDTA (1 mM) used in the above-mentioned studies and in the current study is not detrimental and allows for adequate detection of free amino acids in petiole exudates. In artificial feeding assays, populations of non-viruliferous aphids were reduced on diet supplemented with petiole-exudates from saturated and drought-stressed plants mirroring results observed in whole plants assays. It is hypothesized that drought stress increases amino acid concentration in the phloem sap, but loss of turgor pressure can limit accessibility of phloem sap to aphids thereby reducing population growth (Huberty and Denno, 2004). We found a tendency for greater total free amino acid content in drought-stressed plants and also reduction in feeding duration. Conversely, decrease in amino acid content in saturated plants may have resulted in decrease in aphid populations. Besides changes in amino acid content, drought stress has been shown to enhance sugar content in the phloem which can have positive (Khan et al., 2010; Mewis et al., 2012) or negative (Douglas, 2006) impact on aphid performance. It is plausible that in addition to changes in amino acid composition, water-stress treatments could have caused alterations in the levels of other compounds which have not been evaluated in the study. Future research may be aimed at investigating such compounds using proteomic and metabolomic approaches. In the current study, however, aphids performed poorly on drought-stressed plants which suggests that other changes may outweigh any benefit to the aphid from increased sugar content on drought-stressed plants.

The differential effects of water-stress treatments on growth of aphid populations may be also explained by differences in amino acid profiles. Asparagine and valine are critical for soybean aphid development and fecundity. Soybean aphids reared on diets low in asparagine and valine had longer development times, lower fecundity, and significantly fewer mature into adults (Wille and Hartman, 2008). Conversely, *M persicae* reared on a diet supplemented with asparagine and glutamate displayed enhanced growth (Karley et al., 2002). Moreover, tyrosine, alanine, leucine, and glutamic acid accounted for 43% of variations in the intrinsic rate of increase in populations of the *M. persicae* and *B. brassicae* (Cole, 1997). Proline, one of the markers for drought stress acts as an osmoprotectant in plant cells against water stress. However, we did not observe a significant change in proline concentration under any of the water-stress treatments. The accumulation of proline in soybean plants has been shown to be dependent on the growth stage of the plant and also on the level of drought tolerance (Silvente et al., 2012). In soybean plants, proline accumulation is mainly induced when drought occurs during the flowering and also in genotypes that are less tolerant to drought (Silvente et al., 2012). Furthermore, proline does not effect on aphid population growth rates (Douglas et al., 2001), so it is unlikely to have impacted soybean aphid numbers. To summarize, drought-induced enhancement of total amino acid content in the petiole-exudates did not benefit soybean aphid performance which may, in part, be due to the increase in specific amino acids that were detrimental to soybean aphid growth and development.

Water stress can alter plant’s constitutive and induced defenses against both insect pests and pathogens (Mauch-Mani and Mauch, 2005; Fujita et al., 2006; Asselbergh et al., 2008; Ramegowda and Senthil-Kumar, 2015). We analyzed marker genes associated with various phytohormone signaling pathways in order to elucidate the impact of water stress, insect herbivory and virus transmission on plant responses. It is well-documented that the phytohormone, ABA is critical in plant response to drought, and, however, our knowledge regarding its functions in response to insects and pathogens is limited (Erb et al., 2012; Pieterse et al., 2012; Biere and Bennett, 2013). The induction of ABA marker genes, *RD20A* and *SCOF1* we observed in uninfested plants under drought stress highlight the importance of ABA. Studies have shown that ABA levels rapidly increased until 7 days and then start to plateau in response to drought stress (Vaseva et al., 2010). In contrast, both ABA-marker genes showed reduced expression under saturated conditions. Flooding can cause reduction in ABA levels due to downregulation by ethylene (Bailey-Serres and Voesenek, 2008). Interestingly, feeding by non-viruliferous aphids significantly increased ABA-marker gene expression under drought stress and well-watered conditions. Recently, it was shown that drought induced the accumulation of transcripts associated with ABA leading to suppression of SA-dependent defenses in *Medicago truncatula* plants that are susceptible to the pea aphid, *Acrythosiphon pisum* (Guo et al., 2016). With respect to soybean and soybean aphid defense response, our findings are in agreement with Studham and MacIntosh (2013) who showed that ABA levels significantly increased in response to soybean aphid feeding at day 7 in a susceptible cultivar. The cultivar, AG3432, used in our study is also a susceptible cultivar suggesting that our results support the hypothesis that a decoy strategy is initiated by aphids to suppress both SA- and JA-mediated defenses.

Salicylic acid signaling pathway is critical for plant resistance against aphids, but JA can also be involved (Goggin, 2007). There is mounting evidence that ABA antagonizes SA through various mechanisms including suppression of SA-inducible defense transcripts (Asselbergh et al., 2008). The suppression of SA-dependent transcripts in drought stressed plants indicates a potential antagonistic interaction between ABA and SA signaling. A corresponding decrease in non-viruliferous aphid numbers observed on saturated plants and increase in numbers in drought-stressed plants further highlights the antagonism between ABA and SA signaling. The effect of ABA on JA on the other hand is more complex with antagonistic and synergistic effects reported (Asselbergh et al., 2008). Similar to the pattern for SA-related genes, the expression of JA marker genes were lowest under drought stress and highest under saturation, which suggests that ABA had a negative impact on JA signaling as well. Taken together, our results suggest that antagonism of ABA on SA and JA is a key element in the interaction between water stress and aphid herbivory.

Plant responses to virus attack is mainly mediated via the SA pathway (Glazebrook, 2005; Koornneef and Pieterse, 2008), hence ABA antagonism of SA can affect plant resistance against virus. For instance, in tobacco plants, infection with the *Tobacco mosaic virus* resulted in an increase in ABA concentration which down–regulated β-1,3- glucanase resulting in increased resistance (Whenham et al., 1986). In the current study, *PR1* expression was highest in response to viruliferous aphid feeding in saturated plants where ABA levels were the lowest. These plants harbored highest amount of virus and transmission rates, suggesting that SA is not critical in virus resistance and there could be other phytohormones involved. We did not, however, find evidence for changes in JA-related genes due to viruliferous aphid feeding under water stress. Transcriptomic analysis of soybean leaf tissue with SMV infection showed that expression levels of many of the transcripts encoding phytohormones were either down-regulated or not affected during early stage of infection (day 7), but upregulated at late stages (day 14 and 21) indicating that plant immune response is not activated until later which may be critical for SMV to establish its systemic infection. (Babu et al., 2008). Hence, future studies may be aimed at analyzing the impact of water stress on virus infection over time.

## Conclusion

This is among the first studies to investigate the effect of drought and saturation on insect herbivory and virus transmission, and the first to undertake a comprehensive analysis of the role of nutrition and defense signaling in plant responses to simultaneous attack by abiotic and biotic stresses. We report that drought and saturation had different consequences for soybean aphids and virus infection and transmission on soybean. Drought and saturation reduced non-viruliferous aphid populations, but had no impact on viruliferous aphids. Nevertheless, virus level and transmission rate was highest in saturated plants and lowest in drought-stressed plants. We were able to show that variation in aphid populations and virus levels correlated with aphid feeding on the corresponding plants. For example, non-viruliferous aphids spent reduced amount of time in SEP on saturated and drought-stressed plants compared to well-watered plants, which presumably resulted in lower populations on these plants. Our findings suggests that plant responses to water stress is complex involving changes in nutrient quality and signaling pathways, which can impact aphid populations and virus transmission. The drought-mediated increase in free amino acid content did not benefit non-viruliferous aphids whereas, a reduction in amino acid content in saturated plants negatively impacted aphid populations. It is possible that quality rather than quantity of specific amino acids had a greater impact on aphid populations. In drought-stressed plants, there was an increase in ABA-related gene expression and decrease in the expression of SA- and JA-related genes compared to saturated plants where the ABA-related gene expression was reduced. These changes in gene expression may in part explain the higher aphid densities on drought-stressed plants compared to saturated plants. Further experimentation including phytohormone analysis and utilizing mutants of the plant defense signaling pathways would be useful to explore this result. Future experiments such as transcriptomic, proteomic and metabolomics approaches may also shed light on specific changes in genes, proteins and metabolites underlying the interaction between water stress, insect herbivory and virus infection.

## Author Contributions

PN and VN conceived and designed the experiments. CC, PSII, JH, and VN performed the experiments. VN and PN analyzed data. PN contributed reagents, consumables, and use of equipment. VN and PN wrote the manuscript.

## Conflict of Interest Statement

The authors declare that the research was conducted in the absence of any commercial or financial relationships that could be construed as a potential conflict of interest.
