# Supplementary figures and images for: Water Stress Modulates Soybean Aphid Performance, Feeding Behavior, and Virus Transmission in Soybean
Source: Front Plant Sci. 2016 Apr 27;7:552. doi: 10.3389/fpls.2016.00552 (PMC4847208; doi:10.3389/fpls.2016.00552)

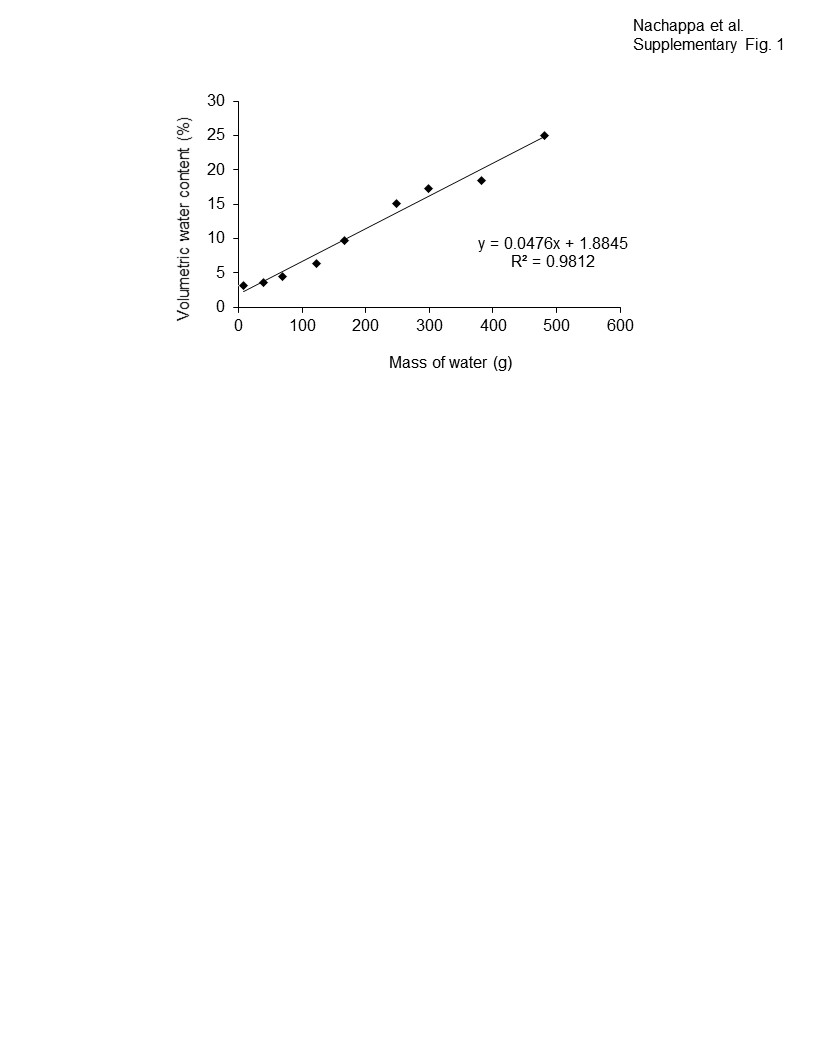

Supplement: Supplementary file 1 [file Image_1.JPEG]

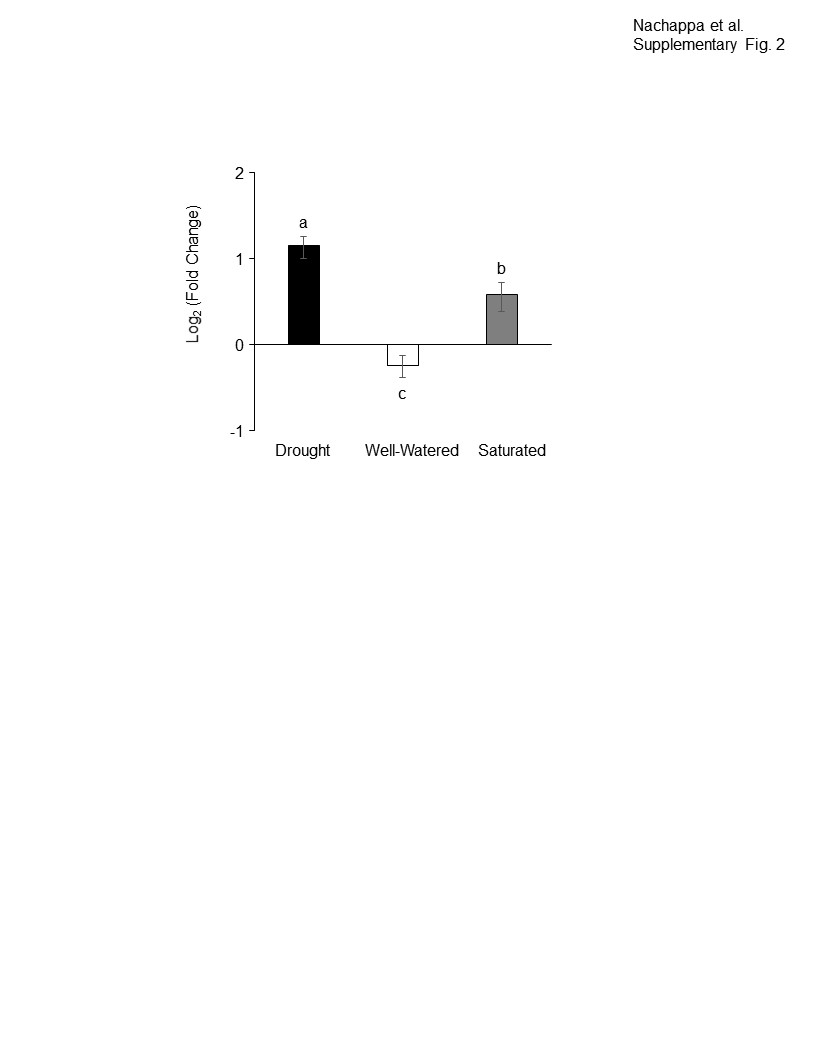

Supplement: Supplementary file 2 [file Image_2.JPEG]

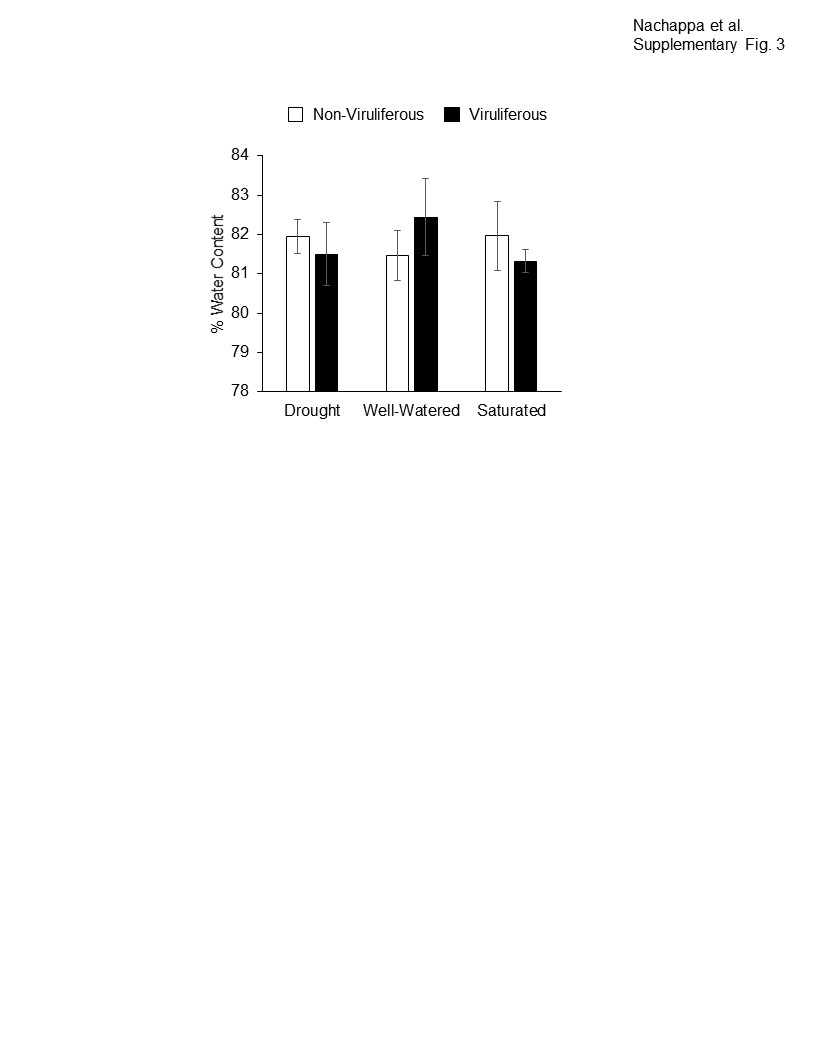

Supplement: Supplementary file 3 [file Image_3.JPEG]
