# Supplementary material for: Water Stress Modulates Soybean Aphid Performance, Feeding Behavior, and Virus Transmission in Soybean
Source: Front Plant Sci. 2016 Apr 27;7:552. doi: 10.3389/fpls.2016.00552 (PMC4847208; doi:10.3389/fpls.2016.00552)
Supplement: Supplementary file 4 [file Data_Sheet_1.DOCX]

**Supplementary Figure 1. Positive correlation exists between volumetric and soil water content.** Regression analysis of average volumetric water content (%) and average mass of water in soilless media (n = 6).

**Supplementary Figure 2. Drought stress causes upregulation of *RD20A*.** Log_2_(fold change) with respect to unstressed controls. Relative gene expression and fold change was calculated using the comparative 2^-ΔΔCT^ method with *FBOX* as endogenous control. Values are shown as mean of log_2_ (fold change) ± SE. Each bar represents the average C_q_ values derived from of n= 9 plants pooled together from three independent experiments. Different letters indicate significant difference between treatments (Tukey’s HSD P<0.001).

**Supplementary Figure 3. Water stress does not affect aphid hydration status.** Percent water content in non-viruliferous and viruliferous aphids collected from plants subjected to drought, well-watered and saturated conditions. Around 30-40 aphids were collected from each plant and pooled for fresh weight and dry weight measurements. Each bar represent the mean ± SE.

Supplementary Table 1. Analysis of variance for aphid population growth in whole plant assays.

| **Source** | **DF** | **F** | ***P-*Value** |
| --- | --- | --- | --- |
| Water Stress | 3 | 18.53 | 0.000 |
| Aphid infestation | 1 | 175.87 | 0.000 |
| Water Stress × Aphid Infestation | 3 | 7.80 | 0.000 |

Supplementary Table 2. Analysis of variance for plant defense signaling gene expression

|  | **Gene** | **Source** | **DF** | **F** | ***P-*Value** |
| --- | --- | --- | --- | --- | --- |
| *ABA* | *RD20A* | Water Stress | 2 | 43.03 | **<0.0001** |
|  |  | Aphid infestation | 2 | 63.38 | **<0.0001** |
|  |  | Water Stress × Aphid Infestation | 4 | 20.59 | **<0.0001** |
|  | *SCOF* | Water Stress | 2 | 31.53 | **<0.0001** |
|  |  | Aphid infestation | 2 | 82.59 | **<0.0001** |
|  |  | Water Stress × Aphid Infestation | 4 | 2.89 | **0.033** |
| *SA* | *PR1* | Water Stress | 2 | 123.20 | **<0.0001** |
|  |  | Aphid infestation | 2 | 413.42 | **<0.0001** |
|  |  | Water Stress × Aphid Infestation | 4 | 24.95 | **<0.0001** |
|  | *PAL2* | Water Stress | 2 | 10.38 | **<0.0001** |
|  |  | Aphid infestation | 2 | 32.26 | **<0.0001** |
|  |  | Water Stress × Aphid Infestation | 4 | 0.23 | 0.92 |
| *JA* | *JAR1* | Water Stress | 2 | 3.17 | **0.05** |
|  |  | Aphid infestation | 2 | 0.30 | 0.74 |
|  |  | Water Stress × Aphid Infestation | 4 | 5.45 | **<0.001** |
|  | *AOS2* | Water Stress |  | 1.11 | 0.338 |
|  |  | Aphid infestation |  | 19.30 | **<0.0001** |
|  |  | Water Stress × Aphid Infestation |  | 1.82 | 0.142 |
